# Superabsorbent cellulose-based hydrogels cross-liked with borax

**DOI:** 10.1038/s41598-022-12688-2

**Published:** 2022-05-26

**Authors:** Supachok Tanpichai, Farin Phoothong, Anyaporn Boonmahitthisud

**Affiliations:** 1 Learning Institute, King Mongkut’s University of Technology Thonburi, Bangkok, 10140 Thailand; 2 Cellulose and Bio-Based Nanomaterials Research Group, King Mongkut’s University of Technology Thonburi, Bangkok, 10140 Thailand; 3 Program of Petrochemical and Polymer Science, Faculty of Science, Chulalongkorn University, Bangkok, 10330 Thailand; 4 Department of Materials Science, Faculty of Science, Chulalongkorn University, Bangkok, 10330 Thailand; 5 Green Materials for Industrial Application Research Unit, Faculty of Science, Chulalongkorn University, Bangkok, 10330 Thailand; 6 Center of Excellence on Petrochemical and Materials Technology, Chulalongkorn University, Bangkok, 10330 Thailand

**Keywords:** Materials science, Soft materials

## Abstract

Cellulose, the most abundant biopolymer on Earth, has been widely attracted owing to availability, intoxicity, and biodegradability. Environmentally friendly hydrogels were successfully prepared from water hyacinth-extracted cellulose using a dissolution approach with sodium hydroxide and urea, and sodium tetraborate decahydrate (borax) was used to generate cross-linking between hydroxyl groups of cellulose chains. The incorporation of borax could provide the superabsorbent feature into the cellulose hydrogels. The uncross-linked cellulose hydrogels had a swelling ratio of 325%, while the swelling ratio of the cross-linked hydrogels could achieve ~ 900%. With increasing borax concentrations, gel fraction of the cross-linked hydrogels increased considerably. Borax also formed char on cellulose surfaces and generated water with direct contact with flame, resulting in flame ignition and propagation delay. Moreover, the cross-linked cellulose-based hydrogels showed antibacterial activity for gram-positive bacteria (*S. aureus)*. The superabsorbent cross-linked cellulose-based hydrogels prepared in this work could possibly be used for wound dressing, agricultural, and flame retardant coating applications.

## Introduction

Hydrogels, three-dimensional porous cross-linked network of macromolecular materials made from hydrophilic polymers with ability to absorb and retain significant quantities of water and resistance to dissolution, have been widely used in many fields such as food, medical, pharmaceutical, agricultural, cosmetic, sensor, and waste treatment. Bio-based hydrogels derived from natural resources including cellulose, hyaluronate, alginate, starch, gelatin, chitin, and chitosan have recently received much attention due to their biodegradability, biocompatibility, and environmental sustainability^1–3^. Among these natural resources, cellulose exhibits a very high yield and mainly derived from plants which are abundant and can be replenished naturally^4,5^.

Water hyacinth (*Eichhornia crasspies*) is an aquatic weed which causes a negative impact on the environment due to their extremely fast growth rate, the impediment to water flow, an enormous water loss through evapotranspiration, and the blocking waterways, disturbing underwater life such as fish and other plants^6–8^. Due to its porous structure and low lignin content (~ 5–9 wt%) compared with other plants, water hyacinth would be an alternative material for cellulose extraction^7,8^.

Prior to forming a hydrogel, cellulose is required to dissolve in a specific solvent or condition to form a liquid solution because cellulose consists of abundant reactive hydroxyl groups (-OH groups) on its structure, leading to the strong intra- and inter-molecular hydrogen bonds forming highly crystalline domains. In this study, the mixture of NaOH/urea solution was selected to dissolve the extracted cellulose from water hyacinth at low temperature due to its low toxicity, quick dissolution time, and low cost^5,9^. The abundant reactive hydroxyl groups (–OH groups) on cellulose molecules lead to high water absorbency and generation of the cross-linked structure. The cross-linking in hydrogels can be generated by three main methods: physical, chemical, and radiation methods. The advantages and drawbacks of each method are listed in Table 1^10–14^.

**Table 1:** Advantages and drawbacks of cross-linking methods to form hydrogels ^10–14^.

Methods	Advantages	Drawbacks
Physical cross-linking	- No use of chemical crosslinking agents - Reversible cross-linking	- Poor stability of hydrogels - Weak cross-linking bonding - Difficulty to control the cross-linking density - Poor mechanical strength
Chemical cross-linking	- Stable hydrogels - Strong cross-linked bonding - High cross-linking density - High mechanical strength	- Toxicity of residual cross-linking agents
Radiation cross-linking	- No requirement for catalysts or additives to initiate a reaction - Possibility to control cross-linking points	- Weak mechanical strength - High cost and energy consumption

Due to the stability and high strength, the chemical cross-linking method was chosen to prepare cellulose hydrogels in this study. However, the main disadvantage of this method is the toxicity of residual cross-linking agents. Therefore, there are many efforts to use low toxic crosslinkers such as carbodiimide^15^, borax^12,16,17^, sodium trimetaphosphate^18,19^, N,N′-methylene bisacrylamide^20^, and polycarboxylic acids^21–23^.

**Figure 1:**
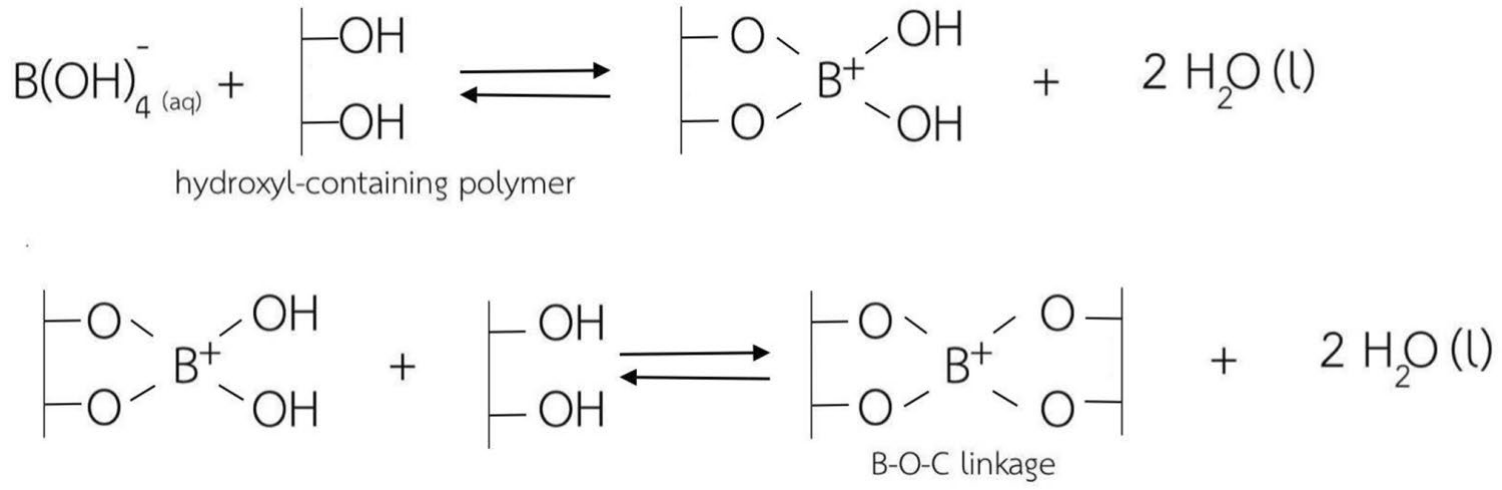
Cross-linking mechanisms between hydroxyl groups of polymers and borax as a cross-linker.

Borax (sodium tetraborate, Na_2_B_4_O_7_.10H_2_O), known as a non-toxic food additive, is an interesting candidate as a chemical cross-linker due to its low toxicity, low cost, and water-soluble ability^12,16,17,24^. Borax in an aqueous solution can be dissociated into trigonal boric acid (B(OH)_3_) and tetrahydroxy borate ion (B(OH)_4_^–^) which are able to react with functional groups of polymers, and then the didiol cross-linking by either covalent or hydrogen bonding is formed^12,17,25–27^, as shown in Fig. 1. The cross-linking of didiol-borax complexes generated by tetrahydroborate ions from borax has been found to be dominated by the pH^28–30^. Increasing the pH of the system, larger amounts of tetrahydroborate ions were generated, and the higher cross-linking reaction could be made from these ions^28^. This resulted in higher mechanical properties (tensile stress and Young’s modulus) of the cross-linked poly(vinyl acetate) films when the films were prepared at a pH of 11 in comparison to those of the cross-linked films prepared at a pH of 4 owing to a higher degree of cross-linking^28^.

Although borax has been successfully used to form cross-linking in various hydrogels such as starch^25^, poly(vinyl alcohol)^12,17^, and guar gum^26^, studies of the use of borax in cellulose hydrogels have been limited. Here, we prepared cross-linked cellulose hydrogels by a dissolution approach using a mixed NaOH/urea solvent (a pH of 11) with aids of borax, and effects of borax concentrations on chemical structure change, crystallinity, thermal stability, morphology, swelling ratio, gel fraction, and antibacterial ability of the cellulose hydrogels were investigated. The utilization of borax as a cross-linking agent would be an environmentally friendly approach to yield the cross-linked cellulose-based hydrogels with high water absorption.

## Experimental

### Materials

Water hyacinth fibers (*Eichornia crassipes*) were obtained from the water hyacinth community enterprise (Baan Pak Tob Chawa) in Phra Nakhon Si Ayutthaya province, Thailand. Ethanol and toluene (Commercial grade, 95% purity) were purchased from CT Chemical Co., Ltd. Analytical-grade Hydrochloric acid (HCl, Conc. 37%) was purchased from JT Baker Chemicals Company, while sodium hydroxide (NaOH, 98% purity) and sodium hypochlorite (NaClO, 4–6% available chlorine) were supplied from Loba Chemie Pvt. Ltd. Urea and borax (> 99% purity) were purchased from Ajax Finechem Pvt. Ltd. and Quality Reagent Chemical Company, respectively. All chemicals and reagents were used as received without further purification.

### Purification of water hyacinth fibers

Water hyacinth fibers were dewaxed with 500 ml mixture solution of toluene-ethanol at 2/1 volume ratio at 75 °C for 3 h. The fibers were subsequently washed and dried at 60 °C for 24 h. The dewaxed water hyacinth fibers were treated by alkaline treatment, bleaching, and acid hydrolysis. 10 g of the fibers was bleached using 3 v/v% NaClO at 80 °C for 2 h, and the bleached fibers were later subjected to alkaline treatment using 1 w/v% NaOH at 60 °C for 2 h to remove hemicellulose. After that, the bleaching process was repeated to obtain purified cellulose, and the purified cellulose was acid-treated using 5 v/v% HCl at 60 °C for 6 h. The treated fibers were washed with distilled water to remove the chemical excess. The treated-cellulose fibers coded as “F” were further used for characterizations and preparation of the cellulose solutions.

### Preparation of cross-liked cellulose-based hydrogels with borax

The cross-linked cellulose-based hydrogels with borax were prepared by dissolving 3 g of dried chemical-treated cellulose fibers into an aqueous solution of 7% NaOH and 12% urea at a low temperature of ~ − 12 °C with vigorous stirring for 3 min to obtain a transparent cellulose solution^31^. After that, the prepared cellulose solution was mixed with various concentrations of borax at room temperature with continuous stirring for 5 min. The weight ratios of cellulose and borax were: 1:0, 1:1, 1:2, 1:3, 1:4 and 1:5. These samples were coded as B0, B1, B2, B3, B4 and B5, respectively. After that, 30 mL of the cellulose-borax solution was poured into a 9 cm-diameter Petri dish and kept at room temperature for 3 days to yield a hydrogel. All hydrogel samples were washed in excess deionized water (DI water) to remove the unreacted components and kept in a plastic container filled with DI water for further characterization.

### Characterization

#### Fourier-transform infrared spectroscopy

Functional group analysis of the water hyacinth fibers, treated cellulose fibers, and cross-linked cellulose-based hydrogels was carried out using a Fourier-transform infrared spectroscope (FTIR) (Nicolet 6700, Thermo Fisher Scientific Co., Lid, USA) in the range from 4,000 to 400 cm^-1^. The dried samples were ground with KBr, and the mixture was pressed into a clear disk for spectrum recording.

#### X-ray diffraction

The crystallinity pattern of the water hyacinth fibers, treated cellulose fibers, and cross-linked cellulose-based hydrogels was determined by x-ray diffraction (XRD) (D8 Advance, Bruker Corp., USA) operated at 40 kV and 40 mA. The samples were scanned over a range of 5 to 60° at 2*θ* with the count step size of 0.5 s and a step size of 0.02°. The crystallinity index was determined with Segal’s empirical method^32^ as followed:

1 $${\text{Crystallinity index }}\left( \% \right) \, = \, \left( {\left( {I_{{{2}00}} {-}I_{{{\text{am}}}} } \right)/I_{{{2}00}} } \right) \times{1}00$$

where *I*_200_ is the maximum intensity of the principal peak of 200 located at 2*θ* = 22°, which represents the crystal part, and the minimum intensity of the peak located at 2*θ* = 16.5° and 22.6°, corresponding to the amorphous part.

#### Thermalgravimetric analysis

The thermal stability of the water hyacinth fibers, treated cellulose fibers, and cross-linked cellulose-based hydrogels was evaluated by thermogravimetric analysis (TGA) (TGA/SDTA 851^e^, Mettler Toledo LLC, USA) under a nitrogen atmosphere with a flow rate of 40 ml min^-1^ at a heating rate of 10 °C min^-1^ over the temperature range of 50–700 °C to investigate the thermal degradation and residue of the samples.

#### UV–vis spectroscopy

The optical transmittance of the cross-linked cellulose-based hydrogels was measured at wavelength from 400 to 700 nm using a UV–Vis spectrophotometer (CE7000, X-Rite, USA). The data were collected at a scan speed of 240 nm min^-1^.

#### Scanning electron microscopy

The cross-section morphological characterization of the cross-linked cellulose-based hydrogels was examined by scanning electron microscope (SEM) (JSM-6610LV, JEOL Ltd., Japan) at an accelerated voltage of 10 kV. The hydrogel samples after swelling in DI water (to an equilibrium swelling) were fractured after lyophilization using a freeze drier (BETA 1–8 LD plus, Martin Christ Gefriertrocknungsanlagen, Germany). Prior to each measurement, the fractured surface of the dried hydrogel sample was sputter coated with a thin layer of gold to prevent charging during the SEM operation.

#### Water content, swelling ratio and gel fraction measurement

The cross-linked cellulose-based hydrogels were immersed in DI water for 24 h to achieve equilibrium swelling. After removal of immoderate surface water, a fully swollen hydrogel was weighted (*w*_s_). Then, the swollen hydrogels were freeze-dried and weighted (*w*_d_) to calculate the water content and swelling ratio according to the following equations:

2 $${\text{water content }}\left( \% \right) = \left( {\left( {w_{{\text{s}}} {-}w_{{\text{d}}} } \right)/w_{{\text{s}}} } \right) \times{1}00$$

3 $${\text{swelling ratio }}\left( \% \right) = \left( {\left( {w_{{\text{s}}} {-}w_{{\text{d}}} } \right)/w_{{\text{d}}} } \right)\times {1}00$$

The gel fraction of the samples was calculated using the following equation.

4 $${\text{gel fraction }}\left( \% \right) = \left( {w_{{{\text{final}}}} /w_{{{\text{initial}}}} } \right) \times{1}00$$

where *w*_initial_ is the weight of the freeze-dried hydrogel, and *w*_final_ is the weight of the dried undissolving part obtained by dissolving the freeze-dried hydrogel into the aqueous solvent of 7% NaOH/12% urea at a temperature of − 12 °C.

#### Antibacterial activity test

The antibacterial activity test of the selected cellulose-based hydrogel was evaluated using the AATCC 100 standard. Prior to characterization, the dried cellulose-based hydrogel film was prepared into 5 × 5 cm. *E. coli* (gram-negative bacteria) and *S. aureus* (gram-positive bacteria) were used as a testing strain to evaluate the antibacterial activity of the hydrogel films. The surface of the films was applied with 10^6^ CFU/ml of those bacterial strains. After that, the prepared films were incubated at 37 °C for 24 h and washed with the fresh broth. The broth that recovered from the specimen was dispersed onto the agar plate an incubated at 37 °C for 24 h. The antibacterial effectiveness of the samples was determined by the percentage reduction of bacteria calculated by the following equation.

5 $$\% {\text{ Reduction of bacteria}} = \left( {\left( {{\text{B }}{-}{\text{ A}}} \right)/{\text{B}}} \right) \times{1}00$$

where A and B are the number of bacteria colonies before and after incubation for 24 h, respectively.

## Results and discussion

### Chemical changes

**Figure 2:**
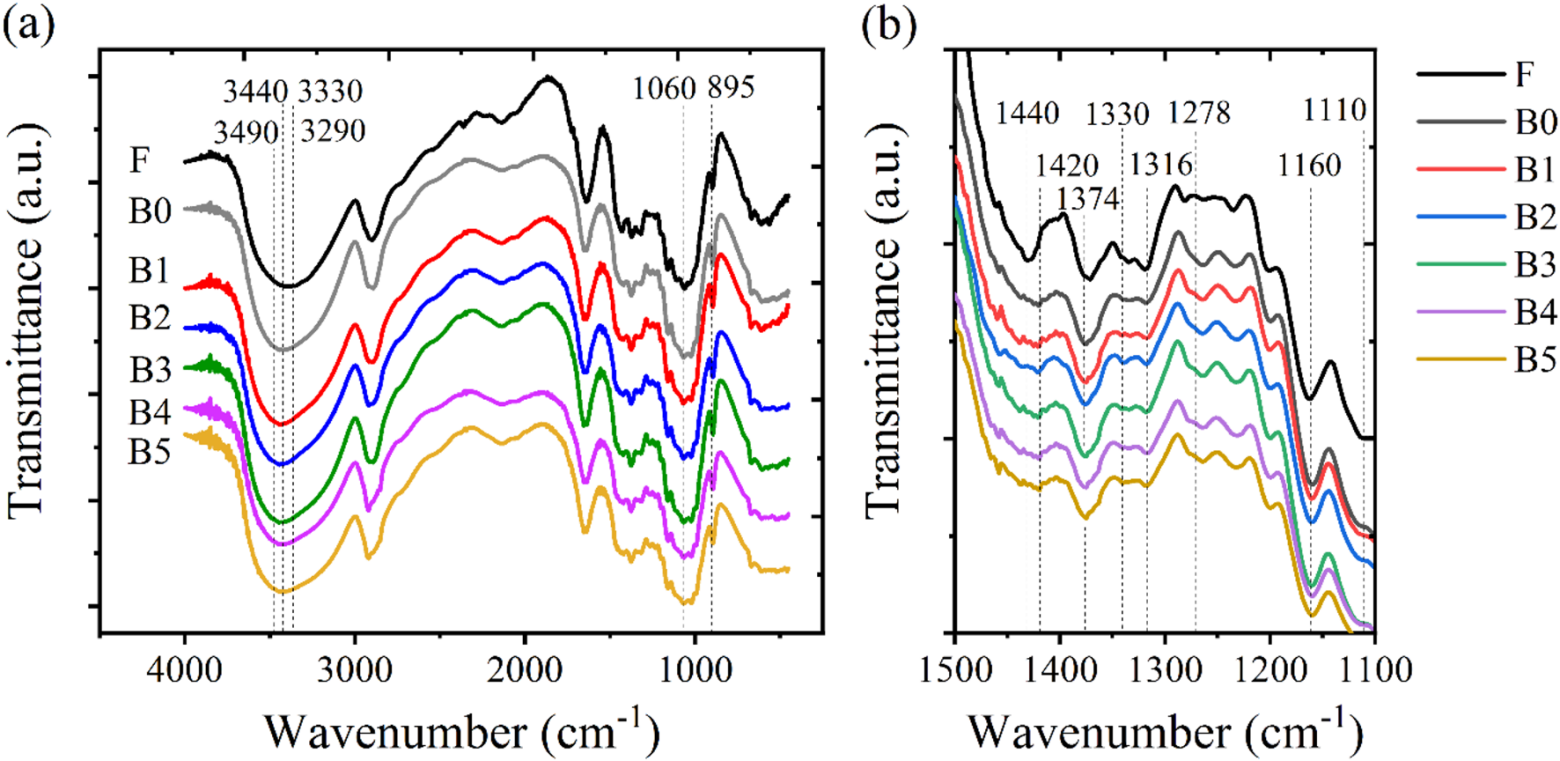
Fourier-transform infrared (FTIR) spectra of purified water hyacinth fibers (F) and cross-linked hydrogels with various concentrations of borax in the range of (a) 4,000 – 400 cm^-1^ and (b) 1,500 – 1,100 cm^-1^.

Effects of dissolution with a mixed NaOH/urea solvent and different loadings of borax on changes in the chemical and crystallographic structure of cellulose were differentiated by FTIR. FTIR spectra of water hyacinth fibers and cross-linked cellulose hydrogels with different borax concentrations are presented in Fig. 2. Purified cellulose fibers have distinguished peaks initially located at 3,330 and 3,290 cm^-1^, corresponding to OH regions generated by O(3)H–O(5) intramolecular hydrogen bonding^33–36^. The typical peaks of cellulose I located at 1,430 cm^-1^ (symmetric CH_2_ bending vibrations), 1,374 cm^-1^ (C–H deformation), 1,316 cm^-1^ (CH_2_ wagging), 1,160 cm^-1^ (symmetric ring stretching), 1,060 cm^-1^ (C–O stretching vibration), and 895 cm^-1^ (asymmetric out-of-plane ring stretching) were observed from these purified cellulose fibers^35–37^. After dissolution in mixed NaOH/urea solvent, the differentiation of these typical peaks was observed in comparison to those of the purified cellulose. It was found that the regenerated cellulose hydrogels presented the cellulose II characteristic with the disappearance or lower intensity of the typical peaks of cellulose I^38^. The flattening of the 3,330 and 3,290 cm^−1^ peak occurred, and two new local maxima appeared at 3,440 and 3,490 cm^−1^. This was associated with an occurrence of a shoulder at 3,130 cm^−1^. The peak located at 1,430 cm^−1^, assigned to CH_2_ stretching of cellulose, shifted to 1,420 cm^−1^ due to changes of the hydroxymethyl group at the C(6) position^33,38,39^, while the band at 1,110 cm^−1^ disappeared^38^. This was an indication of the complete transformation from cellulose I to cellulose II caused by the formation of new intermolecular and intramolecular hydrogen bonding.

With the introduction of borax, there were characteristic peaks of borax and borate occurring in the FTIR spectra at 1,330 and 1,278 cm^−1^, assigned to asymmetric stretching relaxation of B–O–C^16,40,41^. The appearance of these peaks could confirm the formation of the covalent cross-linking network between borate ions and hydroxyl groups of cellulose^27,42^. Also additionally, the hydrogen-bonded crosslinking could be formed between the ring structures of cellulose chain and borax^27^. During the hydrogel preparation, the pH of the cellulose solution prepared with a mixed NaOH/urea solvent was ⁓11, larger amounts of tetrahydroborate ions generated from borax played a vital role in the cross-linking formation. Although the combined cross-linking mechanisms were formed in the system, it would be difficult to measure which cross-linking structure mainly occur^27,42^. Similar formation of complexes between hydroxyl functional groups of poly(vinyl alcohol) and cellulose with aids of borax as a cross-linking agent has been reported^40,43–45^.

### Crystallinity

**Figure 3:**
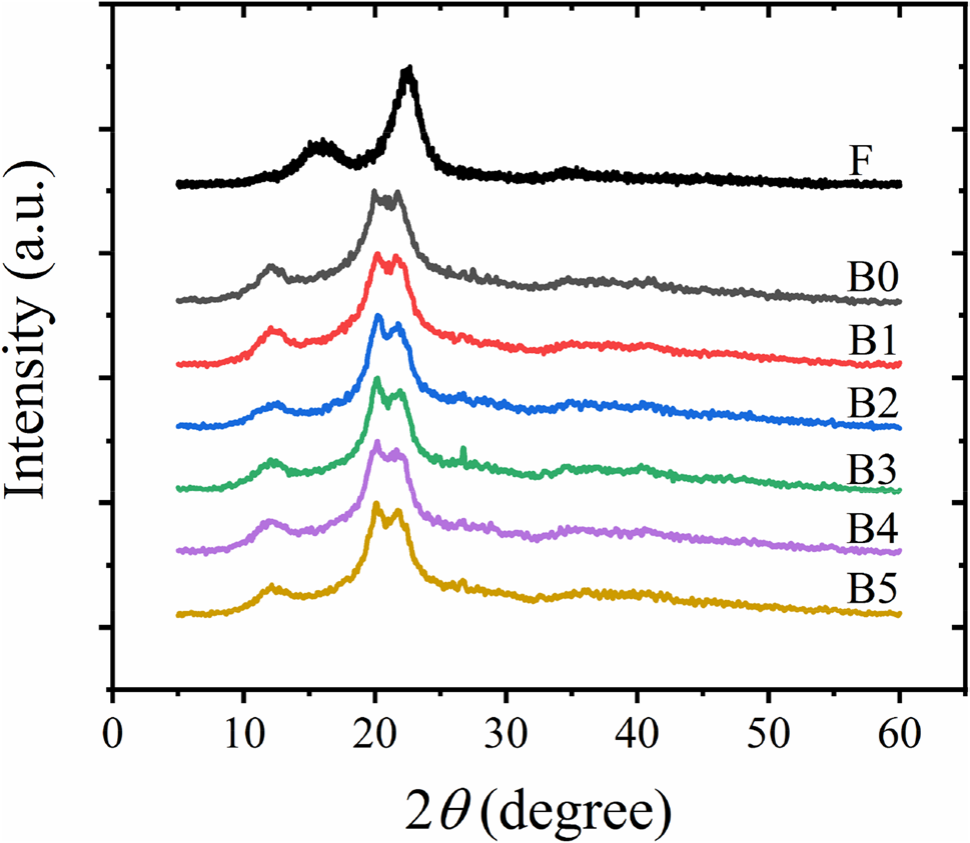
X-ray diffraction (XRD) patterns of purified water hyacinth fibers (F) and cross-linked cellulose hydrogels with various concentrations of borax.

Changes of crystallinity and crystalline forms after dissolution and cross-linking could be differentiated from XRD patterns. Figure 3 presents XRD patterns of the purified fibers and cross-linked cellulose hydrogels with different concentrations of borax. As expected, the cellulose I structure with the peaks located at 14.8, 16.5 and 22.6° lattice planes, corresponding to (1 − 1 0), (1 1 0) and (0 0 2) was observed for the purified water hyacinth fibers^34,39^. This cellulose I structure can be generally found in natural fibers such as cotton^39^, pineapple leaves^46,47^, wood pulp^48,49^, eucalyptus^37^, and abaca^37^. After the water hyacinth fibers were dissolved in a mixed solution of NaOH and urea, the XRD patterns of the regenerated cellulose were transformed from the cellulose I to cellulose II structure with peaks located at 12.0, 20.2 and 22.5°, attributed to (1 − 1 0), (1 1 0) and (0 0 2) lattice planes, respectively. Wei et al.^34^ found that dissolution temperatures of the mixed NaOH/urea solution affected an amount of cellulose I and II in cellulose materials. The mixed solution with a lower temperature could convert a higher amount of cellulose I to cellulose II. The complete disappearance of the peaks related to a cellulose I structure was found when the cellulose was dissolved at temperature of − 12.5 °C, indicating the complete dissolution of cellulose. In this work, the complete conversion of the cellulose structure from cellulose I to cellulose II was observed because the temperature during the dissolution was controlled to be ~ − 12.5 °C. Moreover, the dissolution treatment time is another factor to dominate the conversion of the cellulose structure^48^. The crystallinity index of the water hyacinth fibers was 77.1%, and this crystallinity degree was reduced to 71.1% after regeneration with the mixed solvent of NaOH and urea. This reduced crystalline regions could be owing to the combined mechanisms: the disruption of original inter- and intra-molecular interaction of cellulose molecules by NaOH, the reduction of cellulose aggregates in the cellulose-NaOH complex by urea hydrates, and rearrangement of new hydrogen bond between hydroxyl groups of cellulose in the cellulose-NaOH complex^35,39,50^. A similar reduction in the crystallinity index of regenerated cellulose was reported in comparison with that of original fibers^34,35^.

When various loadings of borax were introduced into the regenerated cellulose hydrogels, no change in the cellulose structure was observed. However, a concentration of borax could increase the percentage of the crystalline regions of the cross-linked regenerated cellulose materials. The uncross-linked hydrogel samples (B0) had a crystallinity index of 70.25%, and with the addition of borax, the crystallinity of the B1 and B3 samples increased to 71.76 and 76.51%, respectively. This increase in crystallinity might be attributed to an increase of bonding between cellulose. However, the crystallinity of the cross-linked B5 hydrogels decreased. The similar reduction in crystallinity was found by Zhang et al.^51^. Borax could destroy intermolecular and intermolecular hydrogen bonding of cellulose in the crystalline area, and B–O–C bonding would be formed between cellulose molecules, and this would restrict the mobility of cellulose chains^41^. Moreover, it was reported that borax could partly change the cellulose structure form cellulose I to cellulose II^51^.

### Thermal properties

**Figure 4:**
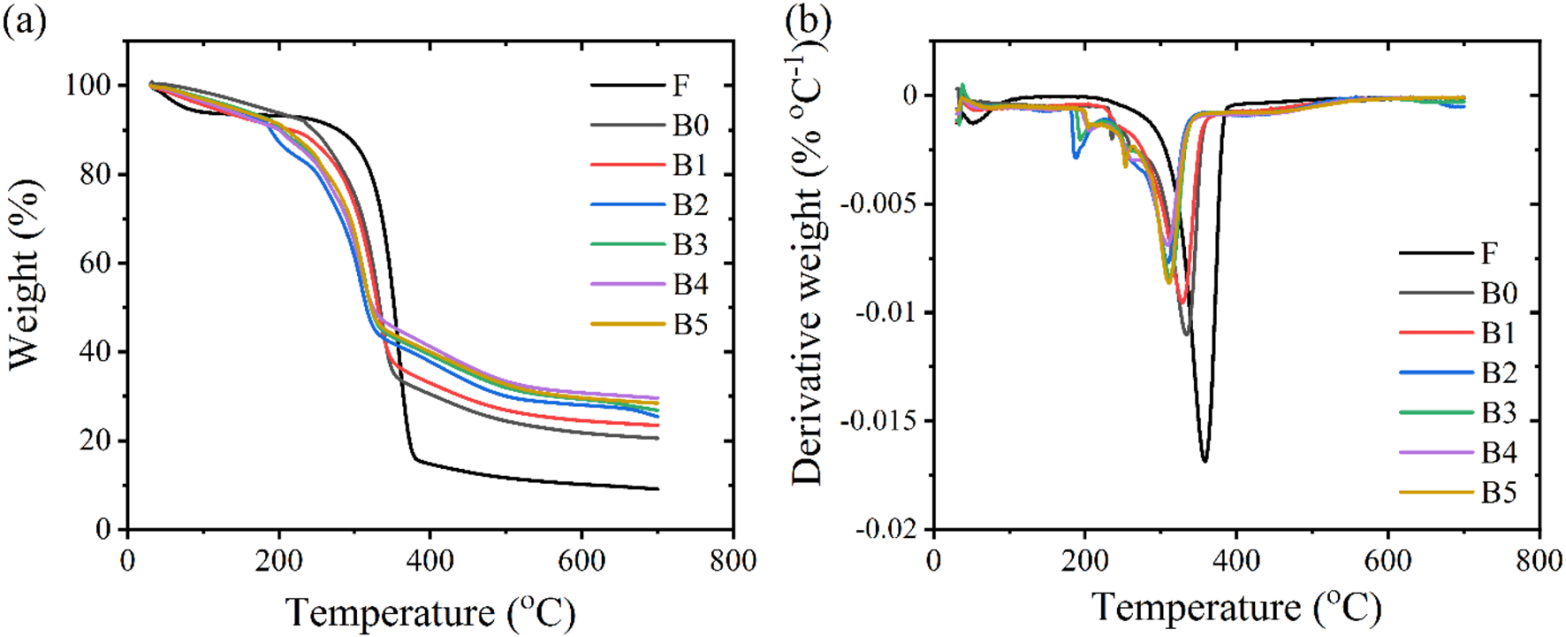
(**a**) Thermogravimetric (TG) and (**b**) derivative thermogravimetric (DTG) curves of purified water hyacinth fibers and cross-linked hydrogels with various concentrations of borax.

Effects of dissolution and cross-linking on thermal stability of cellulose hydrogels were investigated by thermogravimetric analysis (TGA), and the TGA and derivative thermogravimetric (DTG) curves of these cellulose samples are presented in Fig. 4. The initial thermal decomposition state at temperature of less than 100 °C is caused by the moisture evaporation from cellulose materials^52^. The second thermal degradation stage occurs between 250 and 400 °C, corresponding to the thermal degradation of cellulose^53^. It should be noted that there are no thermal transition stages of hemicellulose and lignin found in the curves due to the removal of hemicellulose and lignin by the chemical treatments during the fiber purification step. This resulted in a single peak occurring in a DTG curve. A change in thermal stability of the cellulose materials was noticeable after the dissolution of cellulose fibers. The lower thermal degradation of the B0 materials was observed due to the decomposition of the re-formed crystalline regions (Cellulose II) by the NaOH/urea solvent^34,54^. Moreover, the char content at 700 °C found in the B0 samples was 20.57%, which was significantly higher than that of the cellulose fibers (9.13%). This higher amount of remnants generated from the B0 materials was due to the fact that cellulose II would generate larger amounts of graphite carbon than cellulose I^34^. Additionally, the dissolution temperature affected thermal stability and char formation of the dissolved cellulose^34^. Cellulose dissolved at temperature of − 12.5 °C exhibited lower thermal stability and a larger content of char residues in comparison to those of cellulose materials prepared at the temperature range of 2–10 °C^34^.

With the addition of borax, there are three thermal decomposition states happening. The first thermal decomposition state occurs between 50 and 200 °C owing to the moisture evaporation from borax, and the second transition state is the cellulose degradation which begins between 250 and 350 °C^55–57^. The formation of complexes between cellulose and boron is the final thermal step between 360 and 410 °C^55^. It was found that the onset temperature of cross-linked cellulose hydrogels decreased in comparison to those of uncross-linked hydrogels (B0). This could be attributed to the dehydration of moisture from borax. With increasing borax loadings, a percentage of the weight loss at this stage became greater^54,55^. Borax would play a key role to cause the early thermal degradation^55,56^. Moreover, the maximum thermal decomposition temperature (*T*_max_) of the cross-linked cellulose hydrogels decreased when a larger amount of borax was applied into the hydrogels. *T*_max_ of the B0 material was 339.9 °C, while the addition of borax decreased *T*_max_ of the B1 sample to 330.5 °C. With increasing the borax concentrations, *T*_max_ of the cross-linked cellulose materials was shifted towards a lower temperature. The B5 hydrogels had *T*_max_ of 309.3 °C. This decrease in thermal stabilities of the cross-linked cellulose hydrogels with a higher amount of borax could be due to the esterification of cellulose with borax^55^. A decrease in the DTG peak height was observed when borax was introduced, and the cross-linked cellulose hydrogels with higher contents of borax presented the lower peak height. This reduction could be attributed to thermal annealing of borax and cellulose, which resulted in a slower degradation rate^55^.

The total char formed at 700 °C was found to be dependent on the borax concentration. With the incorporation of borax, the total residue increased from 20.57 (B0) to 23.46% (B1). With a higher content of borax loaded into the cellulose hydrogels, a greater content of the residue was found. The total residue of the B5 sample was 28.45%. The combination of the char formation on cellulose surfaces and generation of water from borax residue and hydroxyl groups of cellulose is an insulator to delay flame ignition and propagation^56,58,59^. This was well-agreed with the fire testing results. The dried B0 sample had a fire growth rate of 1.43 mm s^-1^, while the fire growth rate plummeted to 0.63 mm s^-1^ for the B1 material. This fire growth rate considerably decreased with increasing borax concentrations. The fire growth rate of 0.25 mm s^-1^ was measured from the B5 sample.

### Transparency

**Figure 5:**
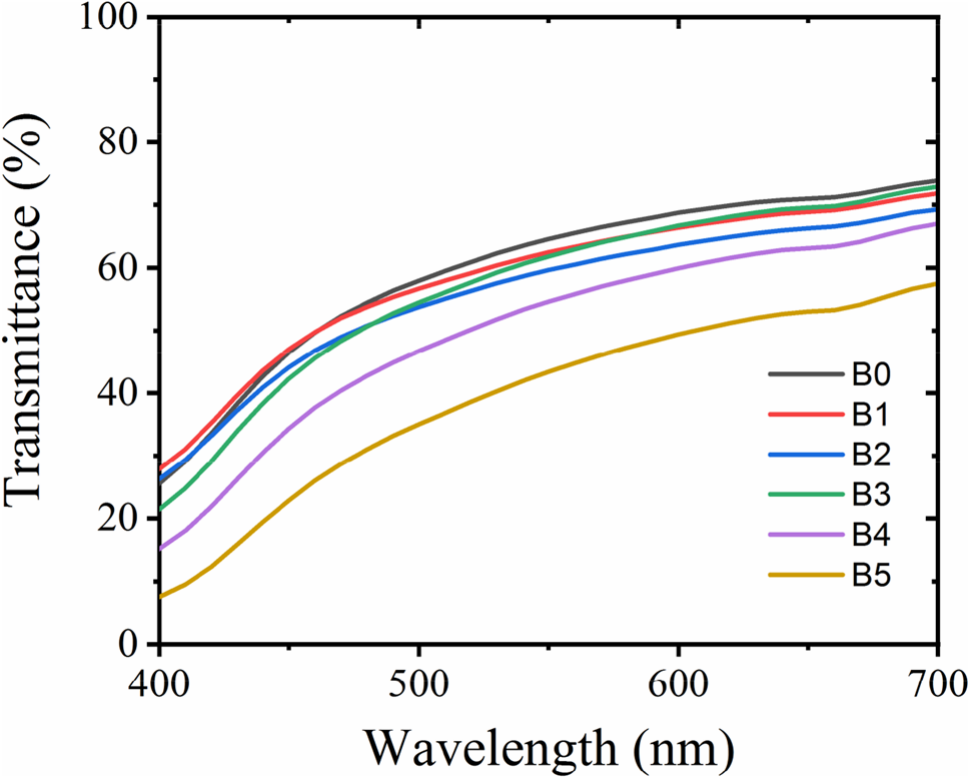
Transmittance of the cross-linked cellulose hydrogels with borax.

Figure 5 presents transparency of the cross-linked cellulose hydrogels with different loadings of borax investigated by UV–vis spectroscopy. 68.8% of the light could pass through the uncross-linked hydrogels (B0) at 600 nm, and with the introduction borax the B1 sample presented a similar transparency to that of B0. However, the transparency of the cross-linked B4 and B5 hydrogels decreased. The transparency of the B5 materials reduced to 49.4% at 600 nm. This might be because more tetrahydroborate ions could from cross-linking in the hydrogels, which blocks or scatters the light. A similar decrease in transparency was reported with the addition of boric acid (BA) into poly(vinyl alcohol) films^41^.

### Morphology

**Figure 6:**
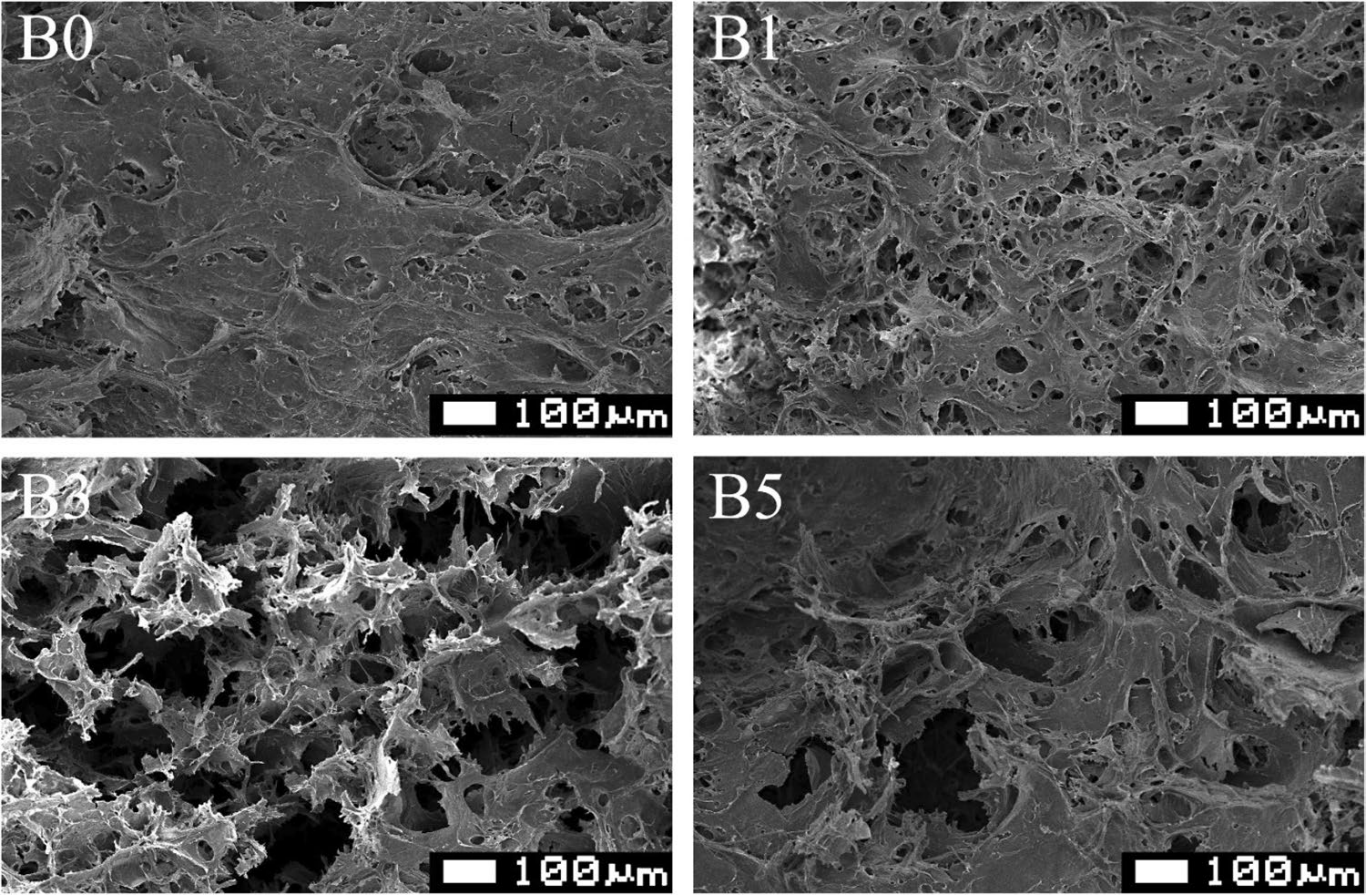
Scanning election microscopy (SEM) images of the cross-linked hydrogels with various concentrations of borax after freeze-drying.

**Figure 7:**
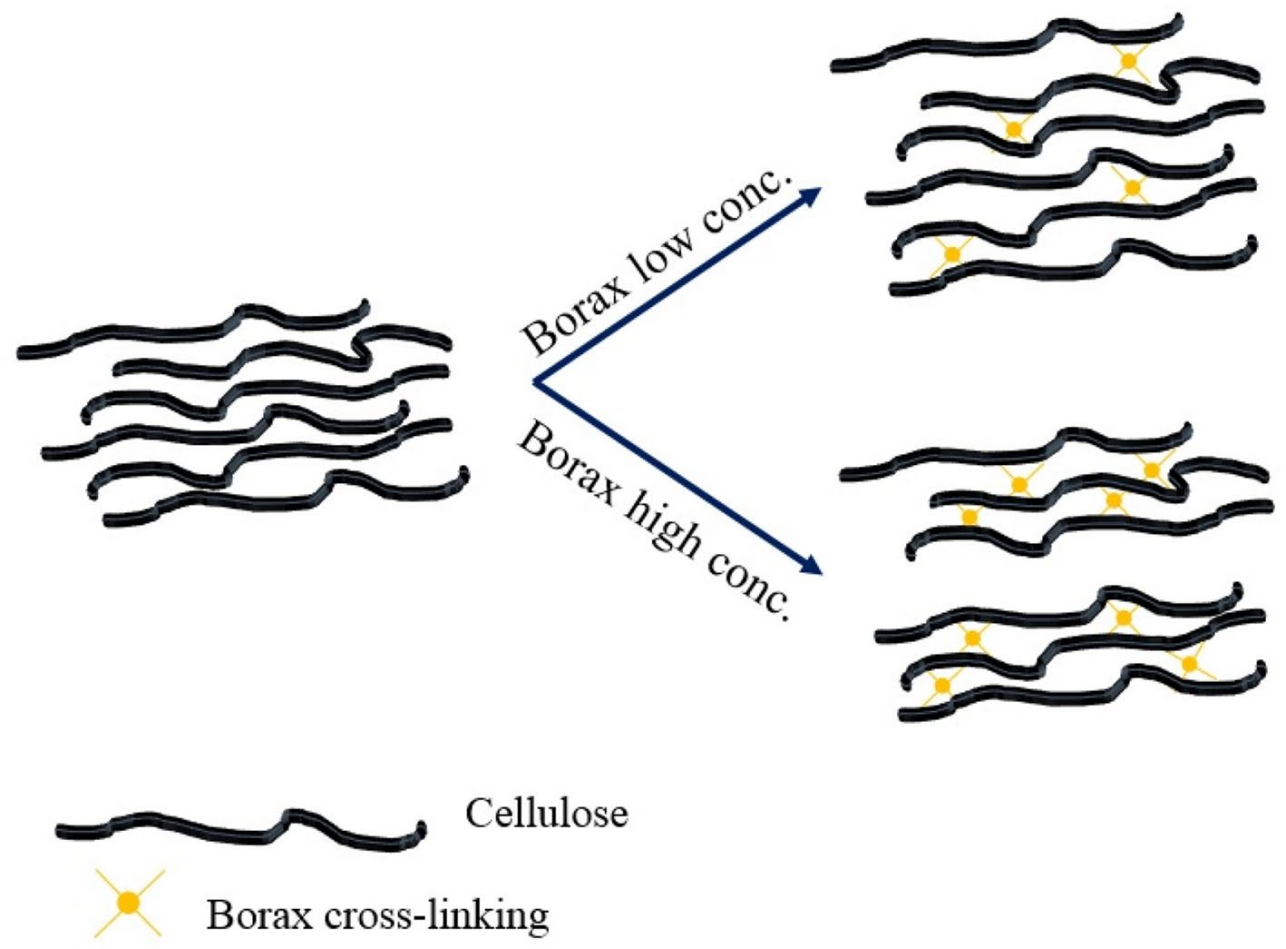
Schematic illustration of cross-linking reaction between cellulose chains at a low and high concentration of borax.

The morphologies of cross-linked cellulose hydrogels with various contents of borax are presented in Fig. 6. The uncross-linked cellulose hydrogel showed a compact structure. This would be due to the interconnection of cellulose chains via hydrogen bonding. A similar compact structure was reported for a carboxymethyl cellulose-chitosan composite hydrogel without any introduction of cross-linkers^60^. On the other hand, with the introduction of borax the porous structure was observed. The B1 hydrogel presented interconnected network with pore diameters of less than 50 µm. This porous structure might be formed by the dense network of cellulose chains with borax. This similar porous structure was also reported for the hydrogels with the addition of citric acid and tartaric acid^60^. The interconnected porous structure was more prominent when the borax concentration was higher. The formation of cross-linking between hydroxyl groups in cellulose chains induced by borax could generate interconnected pore within the dense network structure of cellulose (B3 and B5). This interconnected porous structure induced by borax would affect mechanical properties of the cross-linked cellulose hydrogels. This would be our future work to study mechanical performances of the cross-linked cellulose hydrogels with borax. Figure 7 presents cross-linking reaction between cellulose chains which could generate voids within hydrogels at high loadings of borax.

### Water content, swelling ratio, and gel fraction

The water content in the cellulose hydrogels with borax is presented in Table 2. The uncross-linked cellulose hydrogels (B0) contained water of 90.8%, while with the incorporation of borax, the water content contained in the cross-linked B1 hydrogels increased to 92.6%. When the borax loading was higher, a higher content of water in the cross-linked hydrogels could be obtained. A value of 93.0% for the water content of the B5 hydrogels was reported. This might be because of higher amounts of functional hydroxyl groups generated from borax and cellulose reaction such as monodiol-borax complex interacted with water. It should be noted that the solid content plays a key role to dominate the water content of hydrogels^61^. A hydrogel with a higher solid content would contain less water, compared to a hydrogel with a lower solid content. The water content in the as-prepared hydrogels in this work was similar to that of the hydrogels prepared from poly(vinyl alcohol)^61^.

**Table 2:** Water content, swelling ratio, and gel fraction of the cellulose hydrogels with different loadings of borax. Different letters indicate significant difference (*p *< 0.05) among samples measured under the same testing condition.

Materials	Water content (%)	Swelling ratio (%)	Gel fraction (%)
B0	90.8 ± 0.4^a^	325.2 ± 33.6^a^	2.2 ± 0.3^a^
B1	92.6 ± 0.1^b^	553.1 ± 13.4^b^	8.8 ± 0.2^b^
B2	92.8 ± 0.1^c^	772.4 ± 13.5^c^	10.3 ± 1.5^b^
B3	93.5 ± 0.1^d^	877.3 ± 44.4^d^	17.3 ± 3.1^c^
B4	93.2 ± 0.1^e^	828.2 ± 9.9^d^	18.0 ± 2.6^c^
B5	93.0 ± 0.2^f^	831.0 ± 50.1^d^	21.3 ± 0.5^d^

Significant values are in [a, b, c, d, e, f].

Swelling ratio is the capacity of the dried material to absorb water, which is one of the most important parameters for hydrogel applications^62^. Table 2 presents the swelling ratio of the cross-linked cellulose hydrogels with borax. The uncross-linked cellulose hydrogels (B0) presented the swelling ratio of 325.2%, which was higher than that of poly(vinyl alcohol) hydrogels prepared using a freeze–thaw approach^63^. This higher swelling ratio was attributed to hydroxyl groups on cellulose surfaces which attracted water into the hydrogels^62^. The swelling ratio significantly increased to 553.1% with the introduction of borax. Then, a considerable increase in the swelling ratio was observed for B3 (the swelling ratio of 877%). This increase would be attributed to larger amounts of hydroxyl groups on cellulose molecules generated by borax to form hydrogen bonding with water associated with the porous structure^64^. An increment of the swelling capacity of poly(acrylamide-co-acrylic acid) hydrogels was found with the introduction of spirulina owing to the formation of hydrogen bonding between functional groups on the spirulina surface including hydroxyl, amide, and carboxyl groups^64^. However, no change of the swelling ratio could be observed for the B4 and B5 hydrogels. This might be owing to the limited content of cellulose which could be interacted with tetrahydroborate ions. It was worth noting that the swelling ratio of the prepared cross-linked hydrogels was higher than that of hydrogels previously reported^60,65^. When hydrogels are stimulated by temperature, pH, ionic strength, magnetic field, and radiation, the hydrogel materials would either swell or shrink^66,67^. For example, an increase or decrease in the electrostatic repulsion between polymer chains occurs with a change in environmental pH, resulting in a swell or shrink characteristic of hydrogels^62^. It was reported that the cellulose hydrogels followed the second-order swelling kinetics^62,68,69^. In the initial swelling stage, the hydrogen bonding formed between adjacent cellulose chains limited the swelling of the cellulose networks. While the hydrogen bonds were being broken by water, the expansion of cellulose network was found, and water was accommodated in the cellulose network by osmotic pressure. The swelling capacity of this system achieves the equilibrium asymptotically^68,69^.

The gel fraction was investigated to confirm the cross-linking occurring between tetrahydroborate ions and hydroxyl groups of cellulose. After immersing in the mixed NaOH/urea solvent, only 2.2% of the dried material remained intact, indicating the hydrogen bonding between cellulose chains took place in this uncross-linked hydrogels. However, with the addition of borax, the gel fraction increased to 8.8% for B1. This could be attributed to the occurrence of the cross-linking. With increasing the borax concentrations, a value of the gel fraction increased considerably. B5 presented the gel fraction of 21.3%. This increase resulted from large amounts of tetrahydroborate ions interacted with hydroxyl groups of cellulose. The low value of the gel fraction obtained from the cross-linked cellulose hydrogels prepared in this work might be owing to the lower degree of polymerization and molecular weight of cellulose which limited the mobility of the molecular chains. Moreover, Geng et al.^28^ reported that the gel fraction of the cross-linked poly(vinyl acetate) with borax was mainly controlled by the pH when a concentration of borax was more than a threshold. The gel fraction of cross-linked poly(vinyl acetate) hydrogels with borax prepared at the pH of 11 (~ 70%) was higher than that of the hydrogels prepared at the pH of 9.3 (~ 64%). Therefore, an increase in the pH during the gel preparation step might fabricate hydrogels with a higher degree of cross-linking.

### Antibacterial property

It is known that the cellulose-based hydrogel does not possess antibacterial activity which is the limitation to use in medical applications. Therefore, there are many attempts to introduce particles to improve the antibacterial activity, such as silver nanoparticles, and graphene oxide^23,70^. In this research, it was found that the addition of borax not only produced the cross-linked structure of the cellulose-based hydrogel, but also enhanced its antibacterial activity as shown the percentage reduction of bacteria in Table 3. The B3 hydrogel was chosen for the antibacterial activity characterization. The result showed that the incorporation of borax into cellulose-based hydrogel showed negative results of % reduction of bacteria for gram-negative bacteria (*E. Coli*) testing; it could not destroy gram-negative bacteria. On the contrary, The B3 sample exhibited the antibacterial activity for gram-positive bacteria (*S. aureus)* with 20.7 percent reduction. This might be because the existence of borax was able to destroy the gram-positive bacteria as compared to that of the gram-negative bacteria^71^.

**Table 3:** The percent reduction of *E. coli* bacteria and *S. aureus* bacteria of the cellulose hydrogel with the weight ratios of cellulose and borax of 1 to 3.

Materials	% reduction of *E.coli*	% reduction of *S. aureus*
B3	− 5.11	20.7

## Conclusion

The cellulose solution was initially prepared from water hyacinth fibers with a mixed solvent of urea and NaOH. The transformation from cellulose I to cellulose II was noticed after cellulose dissolution with an aid of the mixed solvent of NaOH and urea. This resulted in a lower degree of crystallinity and weaker thermal stability. Cellulose hydrogels formed by this cellulose solution had a water content of 90.8% and swelling ratio of 325.2%, and gel fraction of this hydrogel is as low as 2.2%. After that, borax was used as a cross-linker to fabricate cross-linked cellulose hydrogels, the swelling ratio and gel fraction increased significantly. Due to cross-linking occurring between tetrahydroborate ions and hydroxyl groups of cellulose, the cross-linked hydrogels could absorb larger amounts of water (900% of the original weight). The architecture of the as-prepared cross-linked hydrogels became porous, while the morphology of the uncross-linked hydrogels was compact. With increasing the borax content, more cavities were connected. The cross-linked hydrogels presented poorer thermal stability with the lower degradation temperature in comparison to the uncross-linked hydrogels. This was due to the esterification of cellulose caused by borax. However, a higher char yield of the cross-linked materials was observed. Moreover, the antibacterial activitiy exhibited the existence of borax effectively destroyed the gram-positive bacteria. Therefore, the prepared cross-linked hydrogels would be useful for superabsorbent wound dressing, agricultural, and flame retardant coating applications.

## Data Availability

The datasets generated during and/or analyzed during the current study are available from the corresponding author on reasonable request.

## References

[1] Bauli CR, Lima GF, De Souza AG, Ferreira RR, Rosa DS (2021). Eco-friendly carboxymethyl cellulose hydrogels filled with nanocellulose or nanoclays for agriculture applications as soil conditioning and nutrient carrier and their impact on cucumber growing. Colloids Surf. A. Physicochem. Eng. Asp..

[2] Pei Z (2021). Self-healing and toughness cellulose nanocrystals nanocomposite hydrogels for strain-sensitive wearable flexible sensor. Int. J. Biol. Macromol..

[3] Boonmahitthisud A, Nakajima L, Nguyen KD, Kobayashi T (2017). Composite effect of silica nanoparticle on the mechanical properties of cellulose-based hydrogels derived from cottonseed hulls. J. Appl. Polym. Sci..

[4] Tanpichai S (2022). Recent development of plant-derived nanocellulose in polymer nanocomposite foams and multifunctional applications: a mini-review. Express Polym. Lett..

[5] Tanpichai S, Boonmahitthisud A, Soykeabkaew N, Ongthip L (2022). Review of the recent developments in all-cellulose nanocomposites: properties and applications. Carbohydr. Polym..

[6] Pakutsah K, Aht-Ong D (2020). Facile isolation of cellulose nanofibers from water hyacinth using water-based mechanical defibrillation: Insights into morphological, physical, and rheological properties. Int. J. Biol. Macromol..

[7] Tanpichai S, Biswas SK, Witayakran S, Yano H (2019). Water Hyacinth: a sustainable lignin-poor cellulose source for the production of cellulose nanofibers. ACS Sustain. Chem. Eng..

[8] Tanpichai S (2021). Extraction of nanofibrillated cellulose from water hyacinth using a high speed homogenizer. J. Nat. Fibers.

[9] Li M-F, Sun S-N, Xu F, Sun R-C (2011). Cold NaOH/urea aqueous dissolved cellulose for benzylation: synthesis and characterization. Eur. Polym. J..

[10] Hu W, Wang Z, Xiao Y, Zhang S, Wang J (2019). Advances in crosslinking strategies of biomedical hydrogels. Biomater. Sci..

[11] Li J, Fang L, Tait WR, Sun L, Zhao L, Qian L (2017). Preparation of conductive composite hydrogels from carboxymethyl cellulose and polyaniline with a nontoxic crosslinking agent. RSC Adv..

[12] Han J, Lei T, Wu Q (2013). Facile preparation of mouldable polyvinyl alcohol-borax hydrogels reinforced by well-dispersed cellulose nanoparticles: physical, viscoelastic and mechanical properties. Cellulose.

[13] Parhi R (2017). Cross-linked hydrogel for pharmaceutical applications: a review. Adv. Pharm. Bull..

[14] Fan L, Yang H, Yang J, Peng M, Hu J (2016). Preparation and characterization of chitosan/gelatin/PVA hydrogel for wound dressings. Carbohydr. Polym..

[15] Lu P-L, Lai J-Y, Ma DH-K, Hsiue G-H (2008). Carbodiimide cross-linked hyaluronic acid hydrogels as cell sheet delivery vehicles: characterization and interaction with corneal endothelial cells. J. Biomater. Sci. Polym. Ed..

[16] Han J (2017). Effects of nanocellulose on the structure and properties of poly(vinyl alcohol)-borax hybrid foams. Cellulose.

[17] Han J, Lei T, Wu Q (2014). High-water-content mouldable polyvinyl alcohol-borax hydrogels reinforced by well-dispersed cellulose nanoparticles: dynamic rheological properties and hydrogel formation mechanism. Carbohydr. Polym..

[18] Leone G (2020). Poly-vinyl alcohol (PVA) crosslinked by trisodium trimetaphosphate (STMP) and sodium hexametaphosphate (SHMP): effect of molecular weight, pH and phosphorylating agent on length of spacing arms, crosslinking density and water interaction. J. Mol. Struct..

[19] Cagnin C, Simões BM, Yamashita F, Andrello AC, De Carvalho GM, Grossmann MVE (2021). Hydrogels of starch/carboxymethyl cellulose crosslinked with sodium trimetaphosphate via reactive extrusion. J. Appl. Polym. Sci..

[20] Ibrahim AG, Elkony AM, El-Bahy SM (2021). Methylene blue uptake by gum arabic/acrylic amide/3-allyloxy-2-hydroxy-1-propanesulfonic acid sodium salt semi-IPN hydrogel. Int. J. Biol. Macromol..

[21] Sotolářová J, Vinter Š, Filip J (2021). Cellulose derivatives crosslinked by citric acid on electrode surface as a heavy metal absorption/sensing matrix. Colloids Surf. A. Physicochem. Eng. Asp..

[22] Lungu A (2021). Nanocellulose-enriched hydrocolloid-based hydrogels designed using a Ca^2+^ free strategy based on citric acid. Mater. Des..

[23] Song S (2021). Antibacterial polyvinyl alcohol/bacterial cellulose/nano-silver hydrogels that effectively promote wound healing. Mater. Sci. Eng. C..

[24] Spoljaric S, Salminen A, Luong ND, Seppala J (2014). Stable, self-healing hydrogels from nanofibrillated cellulose, poly(vinyl alcohol) and borax via reversible crosslinking. Eur. Polym. J..

[25] Sringam J, Trongsatitkul T, Suppakarn N (2020). Effects of borax and montmorillonite contents on mechanical properties of cassava btarch-based composite hydrogels. AIP Conf. Proc..

[26] Thombare N, Jha U, Mishra S, Siddiqui MZ (2017). Borax cross-linked guar gum hydrogels as potential adsorbents for water purification. Carbohydr. Polym..

[27] Itou T, Kitai H, Shimazu A, Miyazaki T, Tashiro K (2014). Clarification of cross-linkage structure in boric acid doped poly(vinyl alcohol) and its model compound as studied by an organized combination of X-ray single-crystal structureanalysis, Raman spectroscopy, and density functional theoretical calculation. J. Phys. chem. B..

[28] Geng S, Haque MM-U, Oksman K (2016). Crosslinked poly(vinyl acetate) (PVAc) reinforced with cellulose nanocrystals (CNC): structure and mechanical properties. Compos. Sci. Technol..

[29] Lu B (2017). One-pot assembly of microfibrillated cellulose reinforced PVA–borax hydrogels with self-healing and pH-responsive properties. ACS Sustain. Chem. Eng..

[30] Lin HL, Yu TL, Cheng CH (2000). Reentrant behavior of poly(vinyl alcohol)–borax semidilute aqueous solutions. Colloid Polym. Sci..

[31] Tang L, Feng Y, He W, Yang F (2019). Combination of graphene oxide with flax-derived cellulose dissolved in NaOH/urea medium to generate hierarchically structured composite carbon aerogels. Ind. Crops Prod..

[32] Segal L, Creely JJ, Martin AE, Conrad CM (1959). An empirical method for estimating the degree of crystallinity of native cellulose using the X-ray diffractometer. Text. Res. J..

[33] Duchemin B, Le Corre D, Leray N, Dufresne A, Staiger MP (2016). All-cellulose composites based on microfibrillated cellulose and filter paper via a NaOH-urea solvent system. Cellulose.

[34] Wei Q-Y (2020). Structure and properties of all-cellulose composites prepared by controlling the dissolution temperature of a NaOH/Urea solvent. Ind. Eng. Chem. Res..

[35] Ghosh I, Haider Q, Sharma C (2019). Characterization of regenerated cellulose prepared from different pulp grades using a green solvent. Cellul. Chem. Technol..

[36] Ou R, Xie Y, Shen X, Yuan F, Wang H, Wang Q (2012). Solid biopolymer electrolytes based on all-cellulose composites prepared by partially dissolving cellulosic fibers in the ionic liquid 1-butyl-3-methylimidazolium chloride. J. Mater. Sci..

[37] Hildebrandt NC, Piltonen P, Valkama J, Illikainen M (2017). Self-reinforcing composites from commercial chemical pulps via partial dissolution with NaOH/urea. Ind. Crops Prod..

[38] Dormanns JW, Schuermann J, Müssig J, Duchemin BJC, Staiger MP (2016). Solvent infusion processing of all-cellulose composite laminates using an aqueous NaOH/urea solvent system. Compos. A: Appl. Sci. Manuf..

[39] Cheng G, Zhu P, Li J, Cheng F, Lin Y, Zhou M (2019). All-cellulose films with excellent strength and toughness via a facile approach of dissolution–regeneration. J. Appl. Polym. Sci..

[40] Ge W, Cao S, Shen F, Wang Y, Ren J, Wang X (2019). Rapid self-healing, stretchable, moldable, antioxidant and antibacterial tannic acid-cellulose nanofibril composite hydrogels. Carbohydr. Polym..

[41] Lim M, Kwon H, Kim D, Seo J, Han H, Khan SB (2015). Highly-enhanced water resistant and oxygen barrier properties of cross-linked poly(vinyl alcohol) hybrid films for packaging applications. Prog. Org. Coat..

[42] Tanpichai S, Oksman K (2018). Crosslinked poly(vinyl alcohol) composite films with cellulose nanocrystals: mechanical and thermal properties. J. Appl. Polym. Sci..

[43] Ding L (2021). Self-healing and acidochromic polyvinyl alcohol hydrogel reinforced by regenerated cellulose. Carbohydr. Polym..

[44] Fan Q (2020). Eco-friendly extraction of cellulose nanocrystals from grape pomace and construction of self-healing nanocomposite hydrogels. Cellulose.

[45] Shao C (2019). Mimicking dynamic adhesiveness and strain-stiffening behavior of biological tissues in tough and self-healable cellulose nanocomposite hydrogels. ACS Appl. Mater. Interfaces.

[46] Tanpichai S, Witayakran S (2018). All-cellulose composites from pineapple leaf microfibers: structural, thermal, and mechanical properties. Polym. Compos..

[47] Tanpichai S, Witayakran S (2017). All-cellulose composite laminates prepared from pineapple leaf fibers treated with steam explosion and alkaline treatment. J. Reinf. Plast. Compos..

[48] Piltonen P, Hildebrandt NC, Westerlind B, Valkama JP, Tervahartiala T, Illikainen M (2016). Green and efficient method for preparing all-cellulose composites with NaOH/urea solvent. Compos. Sci. Technol..

[49] Hu Y, Hu F, Gan M, Xie Y, Feng Q (2021). A rapid, green method for the preparation of cellulosic self-reinforcing composites from wood and bamboo pulp. Ind. Crops Prod..

[50] Qi H, Yang Q, Zhang L, Liebert T, Heinze T (2011). The dissolution of cellulose in NaOH-based aqueous system by two-step process. Cellulose.

[51] Zhang J (2020). Improving lignocellulose thermal stability by chemical modification with boric acid for incorporating into polyamide. Mater. Des..

[52] Arévalo R, Picot O, Wilson RM, Soykeabkaew N, Peijs T (2010). All-cellulose composites by partial dissolution of cotton fibres. J. Biobased Mater. Bioenergy.

[53] Somseemee O, Sae-Oui P, Siriwong C (2021). Reinforcement of surface-modified cellulose nanofibrils extracted from Napier grass stem in natural rubber composites. Ind. Crops Prod..

[54] Zhao D, Liu M, Ren H, Li H, Fu L, Ren P (2013). Dissolution of cellulose in NaOH based solvents at low temperature. Fiber. Polym..

[55] Uddin KMA, Ago M, Rojas OJ (2017). Hybrid films of chitosan, cellulose nanofibrils and boric acid: flame retardancy, optical and thermo-mechanical properties. Carbohydr. Polym..

[56] He S (2019). Bio-inspired lightweight pulp foams with improved mechanical property and flame retardancy via borate cross-linking. Chem. Eng. J..

[57] Hirata T, Werner KE (1987). Thermal analysis of cellulose treated with boric acid or ammonium phosphate in varied oxygen atmospheres. J. Appl. Polym. Sci..

[58] Garba B, Maduekwe AAL (1997). Mechanistic study of sodium tetraborate decahydrate as flame suppressant for wood cellulose. Int. J. Polym. Mater. Polym. Biomater..

[59] Wicklein B, Kocjan D, Carosio F, Camino G, Bergström L (2016). Tuning the nanocellulose–borate interaction to achieve highly flame retardant hybrid materials. Chem. Mater..

[60] Uyanga KA, Daoud WA (2021). Green and sustainable carboxymethyl cellulose-chitosan composite hydrogels: effect of crosslinker on microstructure. Cellulose.

[61] Tanpichai S, Oksman K (2016). Cross-linked nanocomposite hydrogels based on cellulose nanocrystals and PVA: mechanical properties and creep recovery. Compos. A: Appl. Sci. Manuf..

[62] Zainul Armir NA, Mohd Salleh K, Zulkifli A, Zakaria S (2022). pH-responsive ampholytic regenerated cellulose hydrogel integrated with carrageenan and chitosan. Ind. Crops Prod..

[63] Zhang L (2011). High strength graphene oxide/polyvinyl alcohol composite hydrogels. J. Mater. Chem. A..

[64] Aydınoğlu D (2015). Investigation of pH-dependent swelling behavior and kinetic parameters of novel poly(acrylamide-co-acrylic acid) hydrogels with spirulina. E-Polym..

[65] Sheikhi M, Rafiemanzelat F, Moroni L, Setayeshmehr M (2021). Ultrahigh-water-content biocompatible gelatin-based hydrogels: toughened through micro-sized dissipative morphology as an effective strategy. Mater. Sci. Eng. C..

[66] Qin Y, Liu Y, Yuan L, Yong H, Liu J (2019). Preparation and characterization of antioxidant, antimicrobial and pH-sensitive films based on chitosan, silver nanoparticles and purple corn extract. Food Hydrocoll..

[67] Drozdov AD, Declaville Christiansen J (2017). The effects of pH and ionic strength on equilibrium swelling of polyampholyte gels. Int. J. Solids and Struct..

[68] Schott H (1992). Swelling kinetics of polymers. J. Macromol. Sci. B..

[69] Sayyed AJ, Mohite LV, Deshmukh NA, Pinjari DV (2021). Swelling kinetic study with mathematical modeling of cellulose pulp in aqueous N-methyl-morpholine-N-oxide solution. React. Kinet. Mech. Catal..

[70] Barus DA, Humaidi S, Ginting RT, Sitepu J (2022). Enhanced adsorption performance of chitosan/cellulose nanofiber isolated from durian peel waste/graphene oxide nanocomposite hydrogels. Environ. Nanotechnol. Monit. Manag..

[71] Yilmaz MT (2012). Minimum inhibitory and minimum bactericidal concentrations of boron compounds against several bacterial strains. Turk. J. Med. Sci..

